# Cultural adaptation and psychometric validation of the Caring Efficacy scale in a sample of Italian nurses

**DOI:** 10.1371/journal.pone.0217106

**Published:** 2019-05-23

**Authors:** Cesar Ivan Aviles Gonzalez, Maura Galletta, Paola Melis, Paolo Contu, Jean Watson, Gabriele Finco, Maria Francisca Jimenez Herrera

**Affiliations:** 1 Anesthesia and Intensive Care Department, University of Cagliari, Monserrato, CA, Italy; 2 Department of Medical Sciences and Public Health, University of Cagliari, Monserrato, CA, Italy; 3 Pain Therapy Service, University of Cagliari, Cagliari, Italy; 4 Watson Caring Science Institute, Boulder, CO, United States of America; 5 Departament d’Infermeria, Universitat Rovira i Virgili, Tarragona, Spain; Universita degli Studi di Udine, ITALY

## Abstract

Caring is the essence of nursing practice. Caring Efficacy scale was developed with the purpose of measuring nurses’ perceived self-efficacy in orienting and maintaining caring relationships with patients. Since any instruments measuring caring self-efficacy have not been developed in Italy, the study aimed at culturally adapting and validating Caring Efficacy scale in a sample of Italian nurses. A total of 300 registered nurses were asked to fill a self-reported questionnaire; translation-back-translation procedure was carried out to maintain semantic, idiomatic and conceptual equivalence of the original scale. Then, factor analysis was performed in order to test appropriateness of the factor structure. Convergent and discriminant validity was also tested. A two-factor structure with 17 items was found. Results show that Cronbach’s Alpha value was 0.84 for Confidence to Care, and 0.75 for Doubts and Concerns. Correlation analysis for convergent and discriminant validity showed that Confidence to Care was positively correlated with sense of coherence and no significant correlation with Doubts and Concerns was found. Caring efficacy scale can be used by nurse managers as a way of assessing nurses’ self-efficacy and their caring orientation, thus improving quality of patient care.

## Introduction

Caring is the essence of nursing practice and it is based on building an authentic relationship between nurses, patients and patients’ families [1]. Watson’s human caring theory better explains this concept [2–6]. According to Watson, human caring can be defined as the intensity of person-to-person relationship aiming at helping patients to maintain human dignity by achieving a wholeness of mind, body, and soul [5]. Her theory emphasizes the humanity of nursing because it promotes health better than simple medical cures [3]. Non-caring models are more task-oriented than psychological and social aspects; these models can lead to patient dissatisfaction as a main threat to quality care [7]. Watson’s theory was used in different fields of nursing [8,9]. This suggests that it is necessary to address and assess nurses’ caring experience to improve their awareness, as well as their caring outcomes [10].

Caring measures have already been developed and grounded by several studies in the Italian culture (see [11–15]). However, these validated instruments specifically refer to perceived caring behaviour [14,15] and to perceptions related to the dimensions of caring [11,13] in nurses and patients. Although these studies represent an important basis for caring research, any specific instruments providing for the measurement of self-reported caring competence cannot be found in the Italian nursing context. As caring efficacy can both improve patient satisfaction [16] and workers wellbeing [17], measuring nurses’ caring efficacy would allow for the development of strategies that could to reduce any weaknesses, thus helping nurses to better meet the actual needs of hospital patients, as well as to improve their care strategies.

As a result, by following on Watson’s theory, this study aimed at culturally adapting and validating Caring Efficacy Scale in Italy by testing psychometric properties of Coates’s original scale [18].

## Literature review

On the basis of on Watson’s theory and Bandura’s self-efficacy theory [19], Coates [18] developed the Caring Efficacy Scale (CES). CES was developed as a tool for the measurement of nurses’ caring experience, including nurses’ abilities, attitudes and cognitions in establishing a caring relationship. The scale assesses the nurses’ perceived self-efficacy in conveying caring orientation and in reinforcing a therapeutic relationship with patients. In Italian context, several tools measuring human caring are available, such as Care-Italy questionnaire [13] and Caring Behavior Inventory (CBIta). These tools analyse caring by considering different factors: patients’ surveillance, professional relationships with patients, satisfying patients’ and family members’ practical and psychological needs, team consultation, and nurses’ emotions. However, nurses’ caring competencies are not included in these measurements. By taking this factor into account, Coates’ CES appears to be the most appropriated tool. The original scale included 46 items measuring caring attitudes, skills and behaviours by using a six-point Likert scale. The current version totally includes 30 items and is balanced both for positive and negative items. The sense of efficacy in establishing caring relationships with patients is considered as the main factor of the scale. The original scale had a good internal consistency (Cronbach’s Alpha value of .857). Successively, the scale was validated in Chile by Carmen Poblete-Troncoso et al. [20]. The authors found a three-factor structure: the first factor reflected the caring self-efficacy dimension and accounted for the most part of variance. The other two factors did not represent distinct dimensions, but they just captured the inverted items of the tool thus ratifying a one-factor structure of the scale. More recently, in their Australian validation Reid and his colleagues [21] revealed a two-factor structure that correspond to the positively and negatively worded items of the original scale. The two sub-dimensions were called *Confidence to Care*—namely confidence and ability to relate and care for patients—and *Doubts and Concerns*—a person’s uncertainty about his/her ability to relate and care for patients. In addition to Australia and Chile countries, CES has been used in United States [22] and its reliability has been therefore sufficiently demonstrated. Some studies have highlighted positive relationships with clinical competence [18]. Hence, the ability to develop and maintain caring relationships with patients is a crucial aspect in the current nursing practice [23].

## Testing convergent and discriminant validity

The Caring Efficacy Scale was developed on self-efficacy theory [19,24], which refers to beliefs in one’s own capabilities to organize resources and execute courses of action to meet situational demands. In nursing, this sense of control can influence an individual’s ability to build up caring relationships with patients [18]. A recent study in the field has found that self-efficacy is positively correlated to the individual sense of coherence [25]. Sense of coherence (SOC) is an important source of self-efficacy and protection for human health [26]. SOC includes three components: comprehensibility, manageability, and meaningfulness [27]. Comprehensibility represents the cognitive component and can be defined as the extent to which individuals have confidence that life events are understandable and make sense to them. Manageability is the behavioural component, and represents the extent to which individuals have available resources to face situational demands. Meaningfulness is the motivational component and refers to the extent to which individuals are worthy of taking actions on demands, which make sense in their lives from an emotional perspective. However, Antonovsky [26] highlights the indivisibility of the construct. Literature indicated that SOC is a global orientation that conveys a feeling of trust about an individual’s ability to use cognitive, emotional, and instrumental strategies to cope with daily difficulties [28]. Individuals with high levels of sense of coherence better cope with difficult situations and environments [29,30]. Studies in nursing context showed that nurses’ sense of coherence had a positive correlation with professional functioning [31,32]. These findings denote that SOC can be considered as an individual’s self-efficacy measure. By following these premises, it is likely that sense of coherence will be associated with caring efficacy in nurses.

## Study aim

The aim of the study was to culturally adapt, and validate the Caring Efficacy Scale by testing psychometric properties in a sample of Italian nurses.

## Methods

### Setting

The research involved nurses from a University hospital in Southern Italy where students of nursing, medicine and other health care professions are trained. This hospital includes more than 360 beds with more than 400 clinical nurses.

### Procedure

On the basis of Watson’s Human Care theory, we carried out the cultural adaptation of the Caring Efficacy Scale by translation-back-translation procedure [33]. Guidelines for the translation, adaptation and validation of the scale were also followed [34] to maintain semantic, idiomatic and conceptual equivalence of the original questionnaire. Two bilingual linguistic experts and two Italian nurse academics translated the original questionnaire independently, from English into Italian. The translations were then compared and the main inconsistences were identified and discussed. Some sentences were linguistically and culturally adapted After reviewing, a unique Italian version of the questionnaire was created. Then, the Italian questionnaire was back-translated into English by another bilingual linguistic expert to assess equivalence. The back-translated version was then compared with the original questionnaire. After comparison, concepts and meanings were considered as understandable and highly equivalent. Once the final version of the Italian instrument was obtained, a pre-test involving 15 nurses was conducted to ensure further validity of the adapted questionnaire. The pre-test assessed quality of the translation, appropriate cultural adaptation, and feasibility of the instrument. Furthermore, it allowed the researchers to assess the time needed to fill in of the questionnaire, which should be within reasonable limits (i.e., 20 minutes) [35]. Nurses were invited to give written recommendations to improve intelligibility of the items as well as of the graphic structure. After pre-test, minimal changes were made to wording and graphic aspects.

### Participants and data collection

After approval from the hospital’s medical Director, convenience sampling was used to select nurses from different wards. All nurses who were interested to take part in the study were recruited. Inclusion criteria were: being clinical nurses, working full-time or part-time in the hospital. Nurse managers were excluded from the survey because they do not have direct relationships with patients. In addition, nursing students were excluded because they are formally not considered as registered nurses.

The Italian version of the full questionnaire was administered in a printed format to 300 nurses in total during their working hours. Data collection was carried out in November 2017; participants were given 3 weeks to complete and return their questionnaire in locked boxes.

### Tools

After obtaining the author’s authorisation, the Caring Efficacy Scale by Coates [18] was used to evaluate the perceived ability to develop caring relationships with patients. The scale included 30 items with a 6-point response Likert- scale going from strongly disagree (−3) to strongly agree (+3).

In addition, the Sense of Coherence scale (SOC) developed by Antonovsky [36] in the validated Italian version by Sardu et al. [37] was used. The aim was to evaluate nurses’ dispositional orientation to face stress and select the most appropriate coping style according to the situation. This version included 13 items with a 7-point Likert- scale.

### Ethical considerations

This study was approved by the Independent Ethics Committee of the Azienda-Ospedaliero Universitaria di Cagliari, Italy. The study conforms to the ethical principles of the Declaration of Helsinki and to the Italian privacy law (Decree No. 196/2003). Written and oral information about the purpose of the study was given to each participant. Participation was voluntary and anonymous. All the nurses were informed that they could leave the survey at any time without consequences for their occupation. To preserve anonymity, verbal informed consent was obtained; the return of the completed questionnaire was considered as informed consent.

### Data analysis

To perform construct analysis, parallel test [38] was used to determine the number of factors of the Caring Efficacy Scale, together with exploratory factor analysis (EFA) and maximum likelihood method with Varimax rotation. In a second step, the obtained factor structure was examined by means of a confirmatory factor analysis (CFA). Alternative models also were tested to assess construct validity.

The fits of the model were assessed by using Comparative Fit Index (CFI) [39], the Tucker Lewis Index (TLI) [40], the Root Mean Square Error of Approximation (RMSEA) [41], and the RMSEA 90% confidence interval [42]. A good fit of the model was obtained when the values for the CFI and TLI were higher of .90 and the RMSEA values close to 0.08 [43,44]. Values smaller than 0.05 for RMSEA 90% confidence interval (CI) indicated very close fit [45]. Akaike’s information criterion (AIC) also was used to compare the models. The model with the lowest AIC value was preferred. Internal consistency of the scale was examined via Cronbach’s alpha (a). Convergent and discriminant validity also was tested by using a self-efficacy measure such as SOC-13. In order to analyse mean values of the subscales, Likert scale values were converted from “-3 to +3” into “1 to 6”.

All the analyses were carried out by using SPSS software program 17.0 (SPSS: An IBM Company, Chicago, IL, USA) and AMOS 18.0 (Chicago, IL, USA).

## Results

### Characteristics of the participants

A total of 300 questionnaires were distributed. The response rate recorded at the end of the missing value analysis was 71.67%. Thus, the total sample included 215 nurses. Approximately 64% (n = 136 of 215) of the sample was women. The average age was 44.3 years (range = 22–63 years, SD = 9.92). About 69.8% (n = 150 of 215) of respondents have been working as nurse for more than 10 years, and 51.6% (n = 111 of 215) of the respondents declared to have a bachelor’s degree. Almost the totality of the sample (89%, n = 191 of 215) have been working full time with a permanent work contract, and 64.7% (n = 139 of 215) have been working on at least two shifts (i.e., morning and afternoon).

### Testing for factor structure and convergent and discriminant validity

In line with Reid et al. [21], parallel test indicated the presence of two factors whose eigenvalues were significantly greater than 1 (p < .05). Item analysis was performed to assess items that provided the best representation of the two dimensions of the Caring Efficacy Scale. We retained items loading on their own reference construct with a factor loading equal or higher than .40. After content analysis, seven items were kept for Confidence to Care sub-dimension. Ten items were kept for the sub-dimension called Doubts and Concerns about nurses’ ability to develop caring relationships with patients. Items 8, 17, 21, 24, and 27 from Doubts and Concerns and items 2, 9, 14, 18, 19, 22, 25, and 28 from Confidence to Care were deleted because their factor loadings were lower than .40, or because they loaded at the same time on different factors. All the final 17 items loaded on their respective factors with factor loading ranging from .40 to .77. The two factors accounted for 35% of the variance. Inter-factor correlation indicated moderate correlations (r = -0.19) between the two factors. Communalities ranged from .23 to .58. The two-factor structure reflected the two mean dimensions of the Caring Efficacy Scale with a shortened measure (see Table 1 for all the items and the factor loadings).

**Table 1:** EFA results: Items and factor loadings for the two-factor structure of Care Efficacy Scale.

		Factor	
Item		Doubt and concerns	Confidence to care
15	I don’t feel strong enough to listen to the fears and concerns of my clients/patients	.774	.182
29	Even when I really try, I can’t get through to difficult clients/patients	.682	.199
26	I often find it difficult to express empathy with clients/patients	.627	.043
16	Even when I’m feeling self-confident about most things, I still seem to be unable to relate to clients/patients	.623	.101
23	If I find it hard to relate to a client/patient, I’ll stop trying to work with that person	.595	.093
13	I feel if I talk to clients/patients on an individual, personal basis, things might get out of control	.583	.090
12	I lack confidence in my ability to talk to clients/patients from backgrounds different to my own	.570	.106
20	I often find it hard to get my point of view across to patients/clients when I need to	.561	.193
1	I do not feel confident in my ability to express a sense of caring to my clients/patients	.490	.215
30	I don’t use creative or unusual ways to express caring to my clients/patients	.442	.085
11	I can usually create some way to relate to most any client/patient	-.266	.682
10	I am able to tune into a particular client/patient and forget my personal concerns	-.287	.682
7	It is easy for me to consider the multi-facets of a clients/patients care, at the same time as I am listening to them	-.153	.595
4	I convey a sense of personal strength to my clients/patients	-.276	.581
3	I feel comfortable in touching my clients/patients in the course of care giving	-.259	.527
6	I have an ability to introduce a sense of normalcy in stressful conditions	-.214	.440
5	Clients/patients can tell me almost anything and I won’t be shocked	-.061	.396
	Cronbach’s Alpha	(.75)	(.84)

*N* = 215

Then, the two dimensions of the shorted version of 17 items were subjected to CFA. The fit of this model was good (Table 2). The two-factor structure of 17 items was compared with two alternative models. The first included (i) a two-factor model with all the 30 items (i.e., 15 items for Doubts and Concerns and 15 items for Confidence to Care dimensions) from the original scale. The second included (ii) a one-factor structure in which all the 17 items from the shorted version loaded into a common factor. The results showed that both the model (i) and model (ii) fitted data significantly worse than the two-factor model with 17 items: Δχ2 (Δdf = 285) = 595.3, p < .001; Δχ2(Δdf = 1) = 219.9, p < .001, respectively (see Table 2 for fit indices).

**Table 2:** Fit statistics of the hypothesized and the alternative models for the Mediterranean countries.

Model	χ2	*df*	CFI	TLI	RMSEA	RMSEA 90% CI	AIC
Two-factor model of 17 items	187.9	116	.925	.912	.054	.039, .068	261.93
Alternative model (i)	783.2	401	.797	.779	.067	.060, .074	911.24
Alternative model (ii)	407.8	117	.698	.649	.108	.097, .120	479.84

p < .05.

Thus, the two-factor structure with 17 items was supported. Cronbach’s Alpha value was 0.84 for Confidence to Care, and 0.75, for Doubts and Concerns. The overall Cronbach’s Alpha for the scale was 0.75. These values are in line with those obtained by Reid et al. [21] and suggest a good reliability for the whole 17-item scale and their sub-dimensions. The mean value reported by nurses was 2.51 (SD = 1.03) for Doubts and Concerns, and 5.05 (SD = .70) for Confidence to Care.

Correlation analysis for convergent and discriminant validity showed that nurses’ SOC was positively correlated with Confidence to Care of the caring efficacy scale (r = .163, p < .05) and there was no evidence for a significant correlation with Doubts and Concerns (r = -.115, p >. 05).

## Discussion

This study aimed at performing cultural adaptation and validation of the Caring Efficacy Scale in a sample of Italian nurses, on the basis of Watson’s Human Caring Theory [2]. The analysis of psychometric properties of the scale appears to be important because good caring relationships can improve patient satisfaction [16] and workers wellbeing [17]. In addition, no instrument measuring nurses’ perceived self-efficacy to convey a caring orientation and develop caring relationships with patients has been developed in the Italian language so far. In line with Reid et al. [21], results show a two-factor structure representing nurses’ Confidence to Care and Doubts and Concerns about their ability to develop caring relationships with patients. Nevertheless, cultural aspects could have influenced the intelligibility of some items. After a content analysis, seven items out of fifteen were kept for the Confidence to Care sub-dimension, and 10 out of fifteen items were kept for Doubts and Concerns. The deleted items (i.e., 8, 17, 21, 24, and 27) for Doubts and Concerns referred to different relationship problems with patients due to personal prejudices, conflicts, and to different culture of patients. Item 8 was too long and difficult to interpret, while items 17 and 27 were too general: they lacked of specific circumstances/problems. Item 21 was ambiguous because it implied that in front of a conflict, nurses did something to solve it. Finally, item 24 referred to relationship problems with patients of a different culture. It is likely that the recruited nurses did not experience similar problems because the percentage rate of foreign patients in the study hospital is less than 3%. As for Confidence to Care sub-dimension, the items 2, 9, 22, and 25 were deleted because they provided a description of nurses’ experience of caring that could not be representative of all the working situations. In effect, in some hospital units, nurses have a limited time to interact with patients because of a brief hospitalization. Items 18 and 19 were also removed because of redundancy. Finally, items 14 and 28 referred to communication aspects with patients, but they were too vague and were deleted from analysis. As expected, nurses declared to have low doubts and concerns about caring orientation and relationship with patients, and reported a high average value for Confidence to Care. These findings indicate that the two subscales are in line with the theoretical conceptualization of the construct, while supporting the results of Reid et al. [21].

The shortened version of 17 items provides for a better representation of the Caring Efficacy Scale in the Italian nursing context. In addition, the advantage of a reduced version allows for the study of the whole construct without making a questionnaire that is too long [46], thus reducing the time needed to complete it, as well as reducing potential non-response rates for participants.

Overall, the 17 items of the Caring Efficacy Scale constitute a robust measure of caring orientation displaying a Cronbach’s Alpha value exceeding the .70 [47] both for the two sub-dimensions and the whole scale. On the basis of these results, there are two distinct factors that best capture caring orientation in Italian nurses, as also shown by the Australian results [21].

As expected, the Caring Efficacy scale appeared to be correlated with a self-efficacy measure such as SOC. Specifically, SOC and Confidence to Care sub-dimension were positively correlated. This would mean that a nurse’s ability to use cognitive, emotional, and instrumental strategies to cope with professional difficulties [28] increases the likelihood conveying caring orientation and reinforcing a therapeutic relationship with patients [18]. This result is consistent with previous studies in nursing showing that nurses’ SOC has a positive correlation with professional functioning [31,32]. Finally, these findings support the theoretical and empirical assumptions [25,29,30] that suggest that SOC is an important coping source for nurses who have to face difficult situations with patients. SOC and Doubts and Concerns subscale were not significantly correlated. Even though an inverse correlation was expected, overall the results support the convergent and discriminant validity for the instrument.

### Limitations and future research directions

The study has a few limitations to address. Firstly, the sample of this study is limited to one hospital of the southern Italy, thus raising questions regarding generalizability of the results. Secondly, the study sample is rather small if compared with the number of the items. However, we followed Hatcher’s [48] recommendation suggesting a subject to item ratio of at least 5:1 to define an adequate sampling in factor analysis. Thirdly, data collection came from one source (i.e., the nurses) with a single self-administered questionnaire; this could lead to a common method bias.

Finally, another evident issue in this research is the use of only one positive construct (SOC) related to the sub-dimensions of caring efficacy, which is considered as an antecedent variable. This limits the possibility to test significant correlations of the caring efficacy subscales with negative constructs as well, and both with antecedent and consequence variables.

On the basis of the limitations, future studies should expand these results by including more hospitals from the region or from Italian regions in order to further validate the current structure of the scale. Also, the scale validity should be tested by comparing different groups of nurses who work in different nursing settings. In addition, future research should analyse the scale stability via longitudinal data or by other sources such as head nurse, and compare these results with data from existing well-validated measures [21].

Future research actions could seek to verify the association of the caring efficacy scale with negative outcomes such as stress, that is commonly found in nursing settings [49], but also turnover and absenteeism. Finally, the effects of the subscales with organizational aspects (i.e., delay in discharge), patients’ satisfaction, and/or quality of care could be investigated. The study of the relationship of caring efficacy with these additional variables would further increase the confidence in the predictive validity of the scale.

## Conclusion

The caring efficacy scale can be used to identify the main aspects that may affect the caring orientation of nurses and use this information to address education and training. Therefore, this tool can become a valuable resource for nurse managers that can efficiently measure the self-efficacy of the nurse on the basis of the human caring theory, thus identifying any weaknesses in providing care in order to improve patient’s health. In addition, this instrument can also be useful for future nurses [22] during clinical learning to improve the critical aspects of the therapeutic approach to the patient. According to Reid et al. [21], the scale can examine the effects of counselling programs and professional development to improve self-efficacy in nurses in health institutions.

## Supporting information

S1 File Caring Efficacy Scale (English and Italian versions). (DOCX)

S1 Dataset (SAV)
