# Supplementary material for: Cultural adaptation and psychometric validation of the Caring Efficacy scale in a sample of Italian nurses
Source: PLoS One. 2019 May 23;14(5):e0217106. doi: 10.1371/journal.pone.0217106 (PMC6532878; doi:10.1371/journal.pone.0217106)
Supplement: S1 File — (DOCX) [file pone.0217106.s001.docx]

| CARING EFFICACY SCALE | |
| --- | --- |
| ITALIAN | ENGLISH |
| 1 Non mi sento fiducioso/a nella mia capacità di esprimere un senso di cura verso i miei pazienti/clienti | 1 I do not feel conﬁdent in my ability to express a sense of caring to my clients/patients. |
| 2 Se non mi sento ben relazionato/a con il mio paziente/cliente, provo ad analizzare cosa posso fare per entrare in contatto con lui | 2 If I am not relating well to a client/patient, I will try to analyze what I can do to reach him/her. |
| 3 Mi sento a mio agio a toccare i miei pazienti/clienti durante l'assistenza. | 3 I feel comfortable in touching my clients’ patients in the course of care giving. |
| 4 Trasmetto un senso di forza personale ai miei pazienti/clienti | 4 I convey a sense of personal strength to my clients/patients. |
| 5 I pazienti/clienti possono dirmi qualsiasi cosa e non sarò scandalizzato/a. | 5 Clients/patients can tell me almost anything and I won’t be shocked. |
| 6 Ho la capacità di mostrare un senso di normalità in condizioni di stress. | 6 I have an ability to introduce a sense of normalcy in stressful conditions. |
| 7 É facile per me prendere in considerazione i molteplici aspetti della cura dei pazienti/clienti e, allo stesso tempo, ascoltarli | 7 It is easy for me to consider the multi-facets of a clients/patients care, at the same time as I am listening to them. |
| 8 Ho difficoltà a mettere da parte i miei pregiudizi e convinzioni personali al fine di ascoltare e accettare un paziente/cliente come persona | 8 I have difﬁculty in suspending my personal beliefs and biases in order to hear and accept a client/patient as a person. |
| 9 Posso entrare in una stanza con un portamento di energia e serenità che faccia sentire pazienti/clienti meglio | 9 I can walk into a room with a presence of serenity and energy that makes clients/patients feel better. |
| 10 Sono in grado di entrare in sintonia con un particolare paziente/cliente e dimenticare le mie preoccupazioni personali. | 10 I am able to tune into a particular client/patient and forget my personal concerns. |
| 11 Di solito riesco a creare un modo di relazionarmi con qualsiasi paziente/cliente | 11 I can usually create some way to relate to most any client/patient. |
| 12 Mi manca la fiducia nella mia capacità di parlare con i pazienti/clienti provenienti da ambienti diverso dal mio. | 12 I lack conﬁdence in my ability to talk to patients from backgrounds different to my own |
| 13 Ho la sensazione che se parlo con i pazienti/ clienti su base individuale e personale, le cose potrebbero andare fuori controllo. | 13 I feel if I talk to clients/patients on an individual, personal basis, things might get out of control. |
| 14 Uso ciò che ho imparato nelle conversazioni con pazienti/clienti per fornire assistenza più personalizzata | 14 I use what I learn in conversations with clients/patients to provide more individualized care. |
| 15 Non mi sento abbastanza forte per ascoltare le paure e le preoccupazioni dei miei pazienti/clienti. | 15 I don’t feel strong enough to listen to the fears and concerns of my clients/patients. |
| 16 Anche quando mi sento sicuro/a di me riguardo la maggior parte delle cose, mi pare ancora di non essere in grado di relazionarmi con i pazienti/clienti | 16 Even when I’m feeling self-conﬁdent about most things, I still seem to be unable to relate to clients/patients. |
| 17 Mi sembra di avere problemi nel relazionarmi a pazienti/clienti | 17 I seem to have trouble relating to clients/patients. |
| 18 Di solito stabilisco una stretta relazione con i miei pazienti/clienti | 18 I can usually establish a close relationship with my clients/patients. |
| 19 Di solito riesco a fare in modo di piacere ai pazienti/clienti | 19 I can usually get patients/clients to like me. |
| 20 Spesso è difficile trasmettere il mio punto di vista ai pazienti/clienti quando ne ho bisogno | 20 I often ﬁnd it hard to get my point of view across to patients/clients when I need to. |
| 21 Quando, provo a risolvere un contrasto con un paziente/cliente, di solito peggioro la situazione | 21 When trying to resolve a conﬂict with a patient I usually make it worse. |
| 22 Se penso che un paziente/cliente sia a disagio o possa avere bisogno di aiuto, mi approccio a quella persona | 22 I think a client/patient is uneasy or may need some help, I approach that person. |
| 23 Se trovo difficile stabilire un rapporto con un paziente/cliente, smetto di cercare di lavorare con quella persona. | 23 If I ﬁnd it hard to relate to a client/patient, I’ll stop trying to work with that person. |
| 24 Spesso è difficile relazionarmi con pazienti/clienti con una cultura diversa dalla mia | 24 I often ﬁnd it hard to relate to client/patients from a different culture than mine. |
| 25 Ho aiutato molti pazienti/clienti grazie alla mia capacità di sviluppare relazioni strette e significative | 25 I have helped many clients/patients through my ability to develop close, meaningful relationships. |
| 26 Spesso ho difficoltà ad esprimere empatia verso i pazienti/clienti | 26 I often ﬁnd it difﬁcult to express empathy with clients/patients. |
| 27 Mi capita spesso di essere sopraffatto/a dalla natura dei problemi che i pazienti/clienti stanno vivendo. | 27 I often become overwhelmed by the nature of the problems clients/patients are experiencing. |
| 28 Quando un paziente/cliente ha difficoltà a comunicare con me, io sono in grado di adattarmi al suo livello | 28 When a client/patient is having difﬁculty communicating with me, I am able to adjust to his/her level. |
| 29 Anche quando mi sforzo, non riesco a stabilire un contatto con pazienti/clienti difficili | 29 Even when I really try, I can’t get through to difﬁcult clients/patients. |
| 30 Non uso modi creativi o inusuali per esprimere il senso di cura ai miei pazienti/clienti | 30 I don’t use creative or unusual ways to express caring to my clients/patients. |

For the Sense of Coherence original scale, please refer to Sardu C, Mereu A, Sotgiu A, Andrissi L, Jacobson KM, Contu P. Antonovsky’s Sense of Coherence Scale: Cultural Validation of Soc Questionnaire and Socio-Demographic Patterns in an Italian Population. Clin Pract Epidemiol Ment Health. 2012;8:1-6.

https://www.ncbi.nlm.nih.gov/pmc/articles/PMC3282882/
